# BmooMPα-I, a Metalloproteinase Isolated from *Bothrops moojeni* Venom, Reduces Blood Pressure, Reverses Left Ventricular Remodeling and Improves Cardiac Electrical Conduction in Rats with Renovascular Hypertension

**DOI:** 10.3390/toxins14110766

**Published:** 2022-11-05

**Authors:** Jorge Eduardo Chang Estrada, Keuri Eleutério Rodrigues, Anderson Maciel, Cahy Manoel Bannwart, Wictória Farias Dias, Moisés Hamoy, Russolina Benedeta Zingali, Andreimar Martins Soares, Carolina Heitmann Mares Azevedo Ribeiro, Raquel Fernanda Gerlach, Marta Chagas Monteiro, Alejandro Ferraz Prado

**Affiliations:** 1 Laboratory of Pharmacology and Toxicology of Cardiovascular System, Institute of Biological Sciences, Federal University of Pará, Belém 66075-110, PA, Brazil; 2 Laboratory of Hemostasis and Venoms, Institute of Medical Biochemistry Leopoldo de Meis, Federal University of Rio de Janeiro, Rio de Janeiro 21941-902, RJ, Brazil; 3 Center of Study of Biomolecules Applied in Medicine (CEBio), Oswaldo Cruz Foundation (Fiocruz Rondônia), Federal University of Rondônia, Porto Velho 76812-245, RO, Brazil; 4 Laboratory of Pharmacology and Toxicology of Natural Products, Institute of Biological Sciences, Federal University of Pará, Belém 66075-110, PA, Brazil; 5 São Lucas University Center, Porto Velho 76805-846, RO, Brazil; 6 Laboratory of Hematology, Faculty of Pharmacy, Institute of Health Science, Federal University of Pará, Belém 66075-110, PA, Brazil; 7 Department of Basic and Oral Biology, Faculty of Dentistry of Ribeirao Preto, University of São Paulo (FORP/USP), Ribeirao Preto 14040-904, SP, Brazil; 8 Laboratory of Clinical Immunology and Oxidative Stress, Pharmacy Faculty, Institute of Health Science, Federal University of Pará, Belém 66075-110, PA, Brazil

**Keywords:** cardioprotective effect, remodeling, fibrosis, snake venom, arrhythmia

## Abstract

BmooMPα-I has kininogenase activity, cleaving kininogen releasing bradykinin and can hydrolyze angiotensin I at post-proline and aspartic acid positions, generating an inactive peptide. We evaluated the antihypertensive activity of BmooMPα-I in a model of two-kidney, one-clip (2K1C). Wistar rats were divided into groups: Sham, who underwent sham surgery, and 2K1C, who suffered stenosis of the right renal artery. In the second week of hypertension, we started treatment (Vehicle, BmooMPα-I and Losartan) for two weeks. We performed an electrocardiogram and blood and heart collection in the fourth week of hypertension. The 2K1C BmooMPα-I showed a reduction in blood pressure (systolic pressure: 131 ± 2 mmHg; diastolic pressure: 84 ± 2 mmHg versus 174 ± 3 mmHg; 97 ± 4 mmHg, 2K1C Vehicle, *p* < 0.05), improvement in electrocardiographic parameters (Heart Rate: 297 ± 4 bpm; QRS: 42 ± 0.1 ms; QT: 92 ± 1 ms versus 332 ± 6 bpm; 48 ± 0.2 ms; 122 ± 1 ms, 2K1C Vehicle, *p* < 0.05), without changing the hematological profile (platelets: 758 ± 67; leukocytes: 3980 ± 326 versus 758 ± 75; 4400 ± 800, 2K1C Vehicle, *p* > 0.05), with reversal of hypertrophy (left ventricular area: 12.1 ± 0.3; left ventricle wall thickness: 2.5 ± 0.2; septum wall thickness: 2.3 ± 0.06 versus 10.5 ± 0.3; 2.7 ± 0.2; 2.5 ± 0.04, 2K1C Vehicle, *p* < 0.05) and fibrosis (3.9 ± 0.2 versus 7.4 ± 0.7, 2K1C Vehicle, *p* < 0.05). We concluded that BmooMPα-I improved blood pressure levels and cardiac remodeling, having a cardioprotective effect.

## 1. Introduction

Hypertension is a complex multifactorial disorder characterized by systolic arterial pressure levels of ≥140 mmHg and diastolic arterial pressure of ≥90 mmHg [1]. The incidence of hypertension has increased substantially over the past 30 years, due to lifestyle factors, environmental factors, and population aging [2,3]. In 2010, 1.13 billion people had hypertension globally, about 31% of the world’s population. In addition, hypertension is still the leading risk factor for stroke and myocardial infarction [3].

Hypertension data are most alarming when it is acknowledged that 10–15% of patients have resistant hypertension that requires the use of three different drugs, including an inhibitor of the renin–angiotensin–aldosterone system, a calcium channel blocker, and a diuretic at the highest dose tolerated. In addition, almost 1% of patients have refractory hypertension, and even with the use of multiple drug therapy, there is no decrease in blood pressure [2,3,4,5]. Therefore, there is a growing need to develop new drugs to treat hypertension.

Compounds extracted from snake venoms may be a good alternative for lowering blood pressure [6,7,8,9]. Captopril, obtained from *Bothrops jararaca* venom, belongs to the class of drugs used worldwide to treat hypertension, the Angiotensin Converting Enzyme Inhibitors (ACEI) [6,10,11]. Proteases isolated from the venom of *Trimeresurus mucrosquamatus* can release bradykinin from plasma kininogen in vitro and cleave angiotensin I into an inactive peptide promoting hypotensive action. The BmooPLA2-I peptide, extracted from *B. moojeni*, showed hypotensive activity. However, the mechanisms by which this hypotensive activity occur are not yet well elucidated [7,12].

The metalloproteinase BmooMPα-I is a snake venom metalloproteinase (SVMP), phylogenetically related to ADAM (a disintegrin and metalloproteinase) and ADAMTS (ADAM with thrombospondin motif type 1), belonging to the P-I class, characterized by simpler and shorter domains, presenting only a metalloproteinase domain in its mature form. BmooMPα-I was isolated from the venom of *B. moojeni* and has kininogenase activity, hydrolyzing kininogen releasing bradykinin, a potent vasodilator with hypotensive activity. BmooMPα-I can hydrolyze angiotensin I at post-proline and aspartic acid positions, generating an inactive peptide [13]. Therefore, it reduces the production of angiotensin II, a molecule that increases blood pressure. The effects presented by BmooMPα-I suggest that this molecule may have hypotensive activity. However, BmooMPα-I did not show toxicity in an animal model [14], being described as a non-hemorrhagic, anti-inflammatory and gelatinolytic metalloproteinase [15].

Considering the ability of BmooMPα-I to increase bradykinin, activate kinins and hydrolyze angiotensin I, this study hypothesized that BmooMPα-I has anti-hypertensive activity, thereby improving cardiac remodeling and functioning in a model of renovascular hypertension. Therefore, this study aimed to evaluate the anti-hypertensive action of BmooMPα-I on blood pressure, left ventricle remodeling and cardiac function, in a 2K1C animal model.

## 2. Results

### 2.1. Purification and Activity of BmooMPα-I Protease from B. moojeni Crude Venom

**Figure 1:**
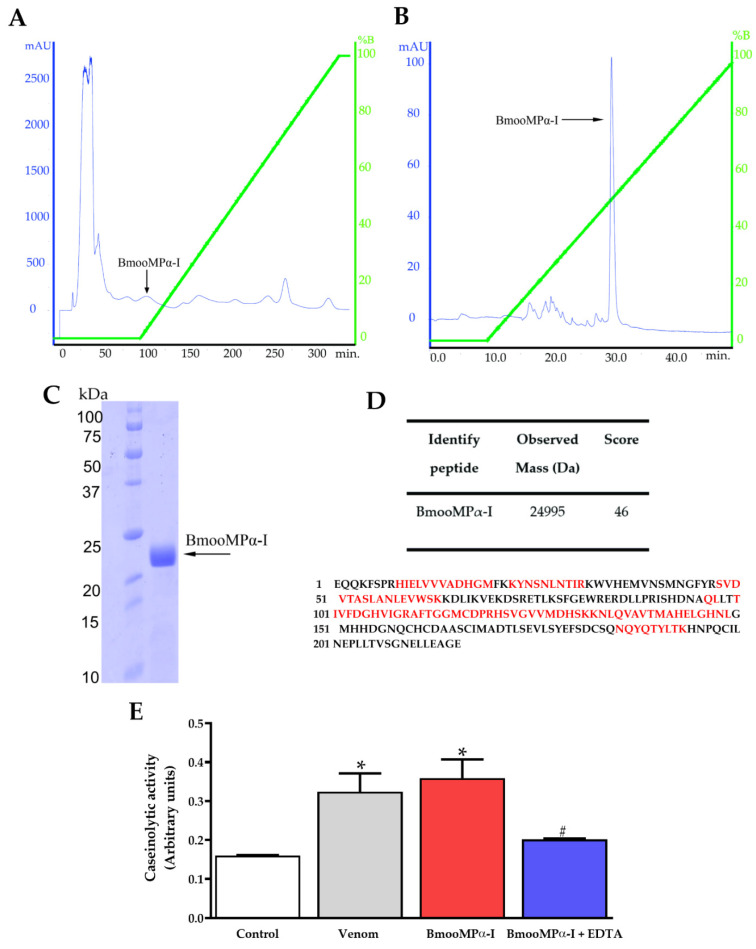
Purification and identification of BmooMPα-I protease extracted from *B. moojeni* venom. (**A**) Fractionation of *B. moojeni* venom (50 mg) by ion-exchange chromatography, the arrow represents the presence of BmooMPα-I before pH inversion with ammonium bicarbonate buffer pH 8.0. (**B**) Reversed-phase column chromatographic profile, the arrow represents the peak corresponding to purified BmooMPα-I. (**C**) Representative Gel SDS-PAGE of the fraction isolated on reversed-phase column showing a single band of the purified protein corresponding to BmooMPα-I. (**D**) Confirmation of the protein isolation by MALDI-TOF. Mascot software and the Swissprot database were used to search for MS/MS ions. The amino acids marked in red represent the amino acids of the isolated protein that matched with the reported sequence of the BmooMPα-I described in databases showing 46% correspondence. (**E**) The BmooMPα-I isolated showed biological activity on the caseinolytic activity assay. Data are presented as mean ± S.E.M. (n = 3/group). * *p* < 0.05 versus control group and ^#^ *p* < 0.05 versus BmooMPα-I group.

The BmooMPα-I protease from *B. moojeni* crude venom was purified by ion-exchange chromatography. Fractionation resulted in six main fractions (Figure 1A), the first peak presented before the pH inversion, corresponding to BmooMPα-I. After purification on an ion-exchange column, the purity of BmooMPα-I was confirmed using p2K1Ce reverse chromatography, where it was possible to observe a single peak, indicating the absence of other proteins in the purified fraction (Figure 1B). The isolated fraction was submitted to polyacrylamide gel electrophoresis (SDS-PAGE), which confirmed a single band of approximately 25 kDa (Figure 1C). The image original of the SDS-PAGE of the fraction isolated can be visualized on supplementary material (Figure S1). MALDI-TOF confirmed the BmooMPα-I in the fraction. Mascot software and the Swissprot database were used to search for MS/MS ions, which revealed a match of 46% correspondence with the reported sequence of the BmooMPα-I described in databases (Figure 1D). The isolated BmooMPα-I showed biological activity on caseinolytic activity assay, while this activity was inhibited in the presence of 20% EDTA (*p* < 0.05, Figure 1E). The purified BmooMPα-I showed similar caseinolytic activity to *B. moojeni* venom (Figure 1E).

### 2.2. Treatment with BmooMPα-I Decreased Systolic Blood Pressure (SBP) and Diastolic Blood Pressure (DBP) in Hypertensive Animals

**Figure 2:**
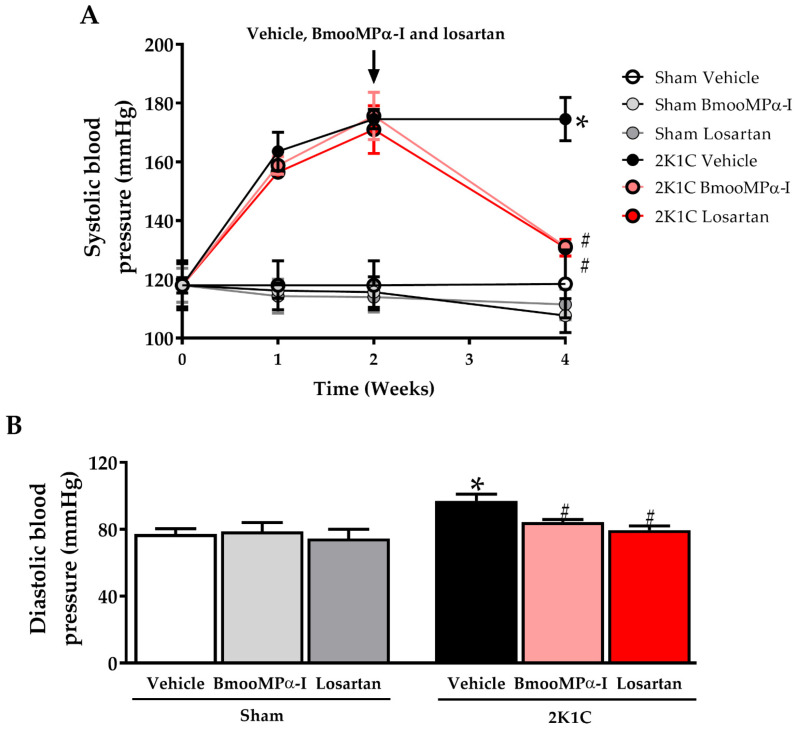
BmooMPα-I and Losartan decreased systolic and diastolic blood pressure in renal hypertensive rats. (**A**) Graphic representation of systolic blood pressure (SBP) in mmHg measured weekly by tail plethysmography for four weeks. The treatment with BmooMPα-I (1 μg/kg), Losartan (10 mg/kg) or Vehicle (water) started in the second week and continued until the fourth week of hypertension. (**B**) Graphic representation of diastolic blood pressure in mmHg measured in the fourth week of hypertension by tail plethysmography. Data are presented as mean ± S.E.M. (n = 6/group). * *p* < 0.05 versus Sham Vehicle group and ^#^ *p* < 0.05 versus 2K1C Vehicle group.

A major goal of this study was to examine whether BmooMPα-I isolated from *B. moojeni* venom had an antihypertensive effect. The 2K1C-hypertension group experienced a sustained increase in blood pressure. In the fourth week of hypertension, the 2K1C group had higher SBP (174 ± 3 mmHg) and DBP (97 ± 4 mmHg), compared to the Sham Vehicle (SBP: 118 ± 4 mmHg and DBP: 77 ± 4 mmHg, *p* < 0.05, Figure 2A,B). The treatment of 2K1C animals with BmooMPα-I reduced the SBP (131 ± 2 mmHg, *p* < 0.05, Figure 2A) and DBP (84 ± 4 mmHg, *p* < 0.05, Figure 2B), with no statistical difference from the 2K1C Losartan group (SBP: 130 ± 3 mmHg and DBP: 79 ± 3 mmHg, *p* > 0.05, Figure 2A,B). No differences were observed between Sham BmooMPα-I (SBP: 110 ± 6 mmHg and DBP: 78 ± 6 mmHg), Sham-Losartan (SBP: 111 ± 6 mmHg and DBP: 74 ± 5 mmHg) and Sham Vehicle (*p* > 0.05, Figure 2A,B).

### 2.3. Treatment with BmooMPα-I Not Changed the Hematological Profile in Sham and 2K1C Animals

Blood was collected to assess the hematological profile to explore the possible toxic implications of BmooMPα-I in the fourth week of normotensive and hypertensive animals. No differences between all groups were observed in the number of erythrocytes, hemoglobin, leukocytes and platelets (*p* > 0.05, Table 1).

### 2.4. Treatment with BmooMPα-I Reversed Electrocardiographic Changes in Hypertensive Animals

The ECG was used to assess possible changes in the frequency and electrical activity of the heart in the fourth week of normotensive and hypertensive animals with BmooMPα-I. Table 2 shows quantitative electrocardiogram data from Sham and 2K1C rats treated with BmooMPα-I, Losartan and Vehicle. The 2K1C Vehicle animals showed electrocardiographic changes, including increased heart rate, prolongation of QRS, QT and QTc interval compared to Sham animals (*p* < 0.05, Table 2). Treatment with BmooMPα-I and Losartan reversed the increase in heart rate and prolonged QRS, QT and QTc intervals, compared to 2K1C Vehicle animals (*p* < 0.05, Table 2). These data were statistically similar to data from Sham animals. No significant changes were observed in the characteristics of the PR interval among all groups evaluated (*p* > 0.05, Table 2).

### 2.5. Treatment with BmooMPα-I Ameliorates the Cardiac Remodeling

**Figure 3:**
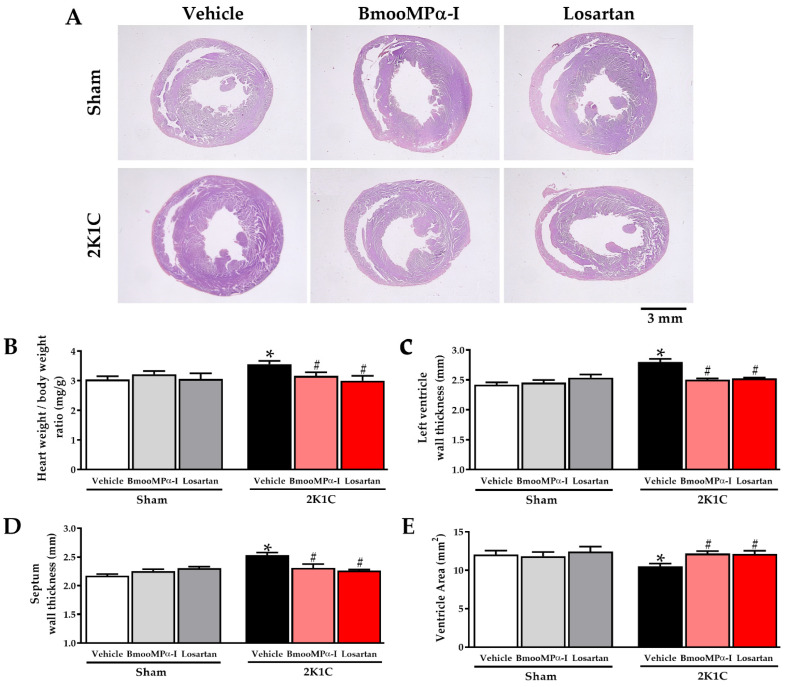
Effects of BmooMPα-I treatment on cardiac morphological alterations induced by hypertension. (**A**) Representative photomicrographs of HE sections (original magnification 10×, representative bar size 3 mm). (**B**) Graph bar of heart weight/body weight ratio. (**C**) Quantification of the left ventricle wall thickness; (**D**) Quantification of the septum wall thickness. (**E**) Quantification of the left ventricle chamber area. Data are shown as mean ± S.E.M. (n = 6/group). * *p* < 0.05 versus Sham Vehicle group and ^#^ *p* < 0.05 versus 2K1C Vehicle group.

Morphometric analyses were performed on the heart using hematoxylin and eosin staining to examine the effects of BmooMPα-I treatment on cardiac remodeling. Figure 3A represents photomicrographs of cross-sections of the heart showing the structural changes in the fourth week of normotensive and hypertensive. The 2K1C hypertension increased the heart weight/body weight ratio, left ventricular wall thickness, interventricular septal thickness, and reduced left ventricular chamber area (*p* < 0.05, Figure 3). The treatment of 2K1C BmooMPα-I animals reduced all histological parameters of cardiac hypertrophy, compared to the 2K1C Vehicle group, with no statistical difference from the 2K1C Losartan group. (*p* < 0.05, Figure 3). There was no statistical difference in any morphometric parameter between all Sham groups analyzed (*p* > 0.05, Figure 3). Table 3 shows quantitative cardiac remodeling data from Sham and 2K1C rats treated with BmooMPα-I, Losartan and Vehicle.

### 2.6. Treatment with BmooMPα-I Decreased the Myocyte Hypertrophy in the Left Ventricle of Hypertensive Animals

**Figure 4:**
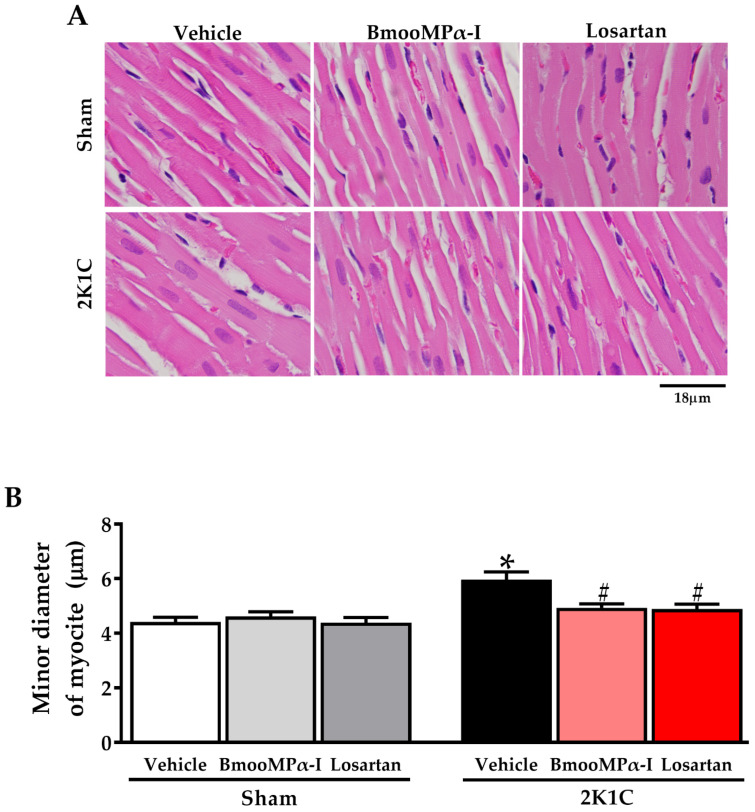
Treatment with BmooMPα-I decreased myocyte hypertrophy in the left ventricle of 2K1C rats. (**A**) Representative photomicrographs of HE sections (original magnification 1000×, representative bar size 18 μm). (**B**) Quantification of myocyte diameter. Data are shown as mean ± S.E.M. (*n* = 6/group). * *p* < 0.05 versus Sham Vehicle group and ^#^ *p* < 0.05 versus 2K1C Vehicle group.

**Figure 5:**
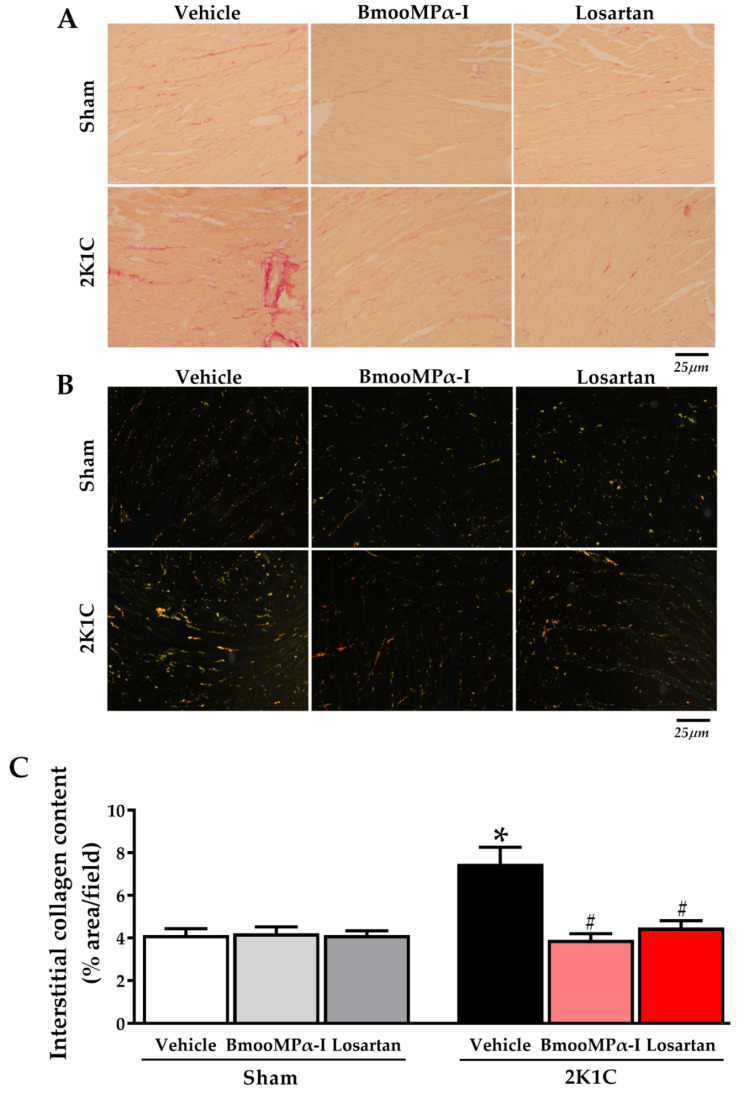
Treatment with BmooMPα-I decreased fibrosis in the left ventricle of 2K1C rats. (**A**) Representative photomicrographs of picrosirius red-stained sections in white light (original magnification 400×, representative bar size 25 μm). (**B**) Representative photomicrographs of picrosirius red-stained sections in polarized light (original magnification 400 x, representative bar size 25 µm). (**C**) Quantification of the collagen content (% area/field) in the left ventricle of the animals. Data are shown as mean ± S.E.M. (n = 5–6/group). * *p* < 0.05 versus Sham Vehicle group and ^#^ *p* < 0.05 versus 2K1C Vehicle group.

Myocyte hypertrophy was evaluated as a parameter of cardiac remodeling. The diameter of myocytes was assessed on slides with HE. The 2K1C-hypertension led to myocyte hypertrophy, compared to the Sham Vehicle (*p* < 0.05, Figure 4). Interestingly, treatment of 2K1C animals with BmooMPα-I decreased myocyte hypertrophy, compared to the 2K1C Vehicle (*p* < 0.05, Figure 4), with no statistical difference with the 2K1C Losartan (*p* > 0.05, Figure 4 and Figure 5). In addition, there was no statistical difference in myocyte diameter between all sham groups analyzed (*p* > 0.05, Figure 4). Table 3 shows quantitative myocyte diameter data from Sham and 2K1C rats treated with BmooMPα-I, Losartan and Vehicle.

### 2.7. Treatment with BmooMPα-I Decreased Interstitial Collagen Content in the Left Ventricle of Hypertensive Animals

Interstitial collagen content was evaluated as a parameter of cardiac fibrosis. Collagen was assessed on slides with red-stained picrosirius. The2K1C-hypertension led to increased interstitial collagen in the left ventricle, compared to the Sham Vehicle (*p* < 0.05, Figure 4 and Figure 5). Interestingly, treatment of 2K1C animals with BmooMPα-I decreased collagen content, compared to the 2K1C Vehicle (*p* < 0.05, Figure 5), with no statistical difference with the 2K1C Losartan (*p* > 0.05, Figure 4 and Figure 5). In addition, there was no statistical difference in collagen content between all Sham groups analyzed (*p* > 0.05, Figure 5). Table 3 shows quantitative collagen content data from Sham and 2K1C rats treated with BmooMPα-I, Losartan and Vehicle.

## 3. Discussion

The main finding of this study was the decrease in blood pressure caused by BmooMPα-I, which was accompanied by improvement in electrocardiographic changes and reversal of left ventricular hypertrophy and fibrosis in animals with renovascular hypertension. In addition, BmooMPα-I, at the dose used, did not change the hematological profile of the animals. This result offers potential as a new anti-hypertensive drug.

The metalloproteinase BmooMPα-I was successfully isolated and purified from *B. moojeni* venom and characterized by mass spectrometry. First, it is essential to highlight that BmooMPα-I showed biological activity similar to that obtained in previous studies [13,14,15]. Another point that should be mentioned is that in our research, the BmooMPα-I and crude venom of *B. moojeni* showed similar caseinolytic activities, probably because other proteases are present in the crude venom, such as serine proteases and other forms of Metalloproteases (PII and PIII) that contribute to this biological activity [16]. However, during protein isolation, these proteases were removed.

Consistent with previous studies, 2K1C hypertension-induced cardiac hypertrophy, increased collagen content, and cardiac dysfunction after two weeks of hypertension [17,18]. Thus, we started treatment with BmooMPα-I when cardiac alterations were already established. This approach helped provide evidence supporting the therapeutic effect of BmooMPα-I as a rescue therapy.

Much of the morbidity and mortality of hypertension is related to its impact on target organs, such as thickening of the heart LV, and fibrosis increases the risk of fatal events, such as acute myocardial infarction [19,20,21,22] and stroke [19]. Therefore, in the clinic, it is always recommended to adopt an antihypertensive regimen capable of decreasing blood pressure and recovering the morphology and function of the myocardial tissue. However, this effect is not consistently seen with anti-hypertensive drugs. For example, 2K1C rats treated with the antihypertensives Zofenopril (6 mg/kg), Nifedipine (30 mg/kg) and Labetalol (40 mg/kg) experienced an effective decrease in blood pressure. However, only Zofenopril and Nifedipine showed benefits in decreasing myocardial fibrosis [23]. Similar blood pressure lowering results, without effects on vascular morphology and biochemistry, were observed in the treatment of 2K1C rats with Aliskiren (50 mg/kg) [24].

Our results with Losartan corroborate previous findings in the 2K1C model, which demonstrated a reduction in blood pressure, myocyte hypertrophy, ventricular wall thickening, and cardiac fibrosis [24,25]. In this study, we compared Losartan and BmooMPα-I to assess whether the effect of BmooMPα-I would be similar to that presented by this drug, which is widely used in the treatment of patients with hypertension. We observed that both drugs had similar effects regarding the reduction of arterial hypertension and cardioprotective effects in 2K1C animals.

Regarding sham animals, treatment with Losartan and BmooMPα-I had no effects on blood pressure and remodeling. These findings were in agreement with previous studies that demonstrated no effects in Sham animals when treated with beta-blockers [26,27], diuretics [28], calcium blockers [29] and AT-1 antagonists [24,25], possibly because these animals do not have a change in blood pressure regulation pathways.

In our results, we verified a reduction in the interstitial collagen content in the left ventricle of the 2K1C BmooMPα-I group, probably related to the ability of BmooMPα-I to cleave angiotensin I, generating an inactive peptide [13], and, thus, reducing the levels of angiotensin II, a TGF-β1 modulating agent that reduces collagen content [18,30]. In addition, BmooMPα-I can release bradykinin [13], which has an anti-fibrotic effect on the myocardium [31,32,33,34,35]. Regarding the proteolytic activity of SVMPs on collagen, a previous protein-protein docking study showed that non-hemorrhagic SVMPs, including BmooMPα-I, cannot cleave collagen [36], thus, suggesting that the reduction of collagen content occurs by the mechanisms mentioned above.

Excessive collagen deposition in the myocardium is identified as one of the causes of poor prognosis, arrhythmias, and premature death in patients with cardiomyopathies [37,38,39,40,41,42]. In addition, fibrosis in the heart impairs the coupling between cardiomyocytes, altering normal electrical conduction [35]. In our study, 2K1C animals showed prolongation of the QRS, QT and QTc intervals, corroborating other studies of hypertension in rats [43,44] and humans [45,46,47].

Previous studies demonstrated an increase in the duration of QRS interval in different models of hypertension in rats [48,49,50,51]. The increased duration of the QRS interval can be explained by a decline in longitudinal conduction velocity in the hypertrophied myocardium [52]. In addition, it has been observed that conduction velocity decreases as the diameter of cardiomyocytes increases [53].

QT and QTc intervals are increased in hypertensive patients with left ventricular hypertrophy. Treatment with antihypertensive drugs propranolol and Losartan reduced ventricular mass associated with partial reversal of arrhythmia and repolarization changes [54]. QT interval prolongation was also described in concentric and eccentric ventricular hypertrophy [55]. In a model of renal hypertensive rats that received the cardiotoxic agent Doxorubicin, an increase in QT and QTc intervals was also demonstrated, associated with increased blood pressure and ventricular hypertrophy. In our study, 2K1C animals showed ventricular hypertrophy with prolongation of the QRS, QT and QTc intervals. Surprisingly, treatment with BmooMPα-I normalized electrocardiographic changes, possibly related to decreased myocyte hypertrophy and collagen volume. Therefore, BmooMPα-I may be an attractive pharmacological tool for treating cardiac remodeling associated with hypertension.

We associate the antihypertensive effect of BmooMPα-I mainly with its kininogenesis activity, thus cleaving kininogen and releasing bradykinin and active kinins [13]. Blood pressure reduction and cardiac hypertrophy by activating the kallikrein–kinin system were demonstrated in 2K1C, DOCA-Salt and spontaneously hypertensive rat models of hypertension by injection of a construct containing the human kallikrein gene [56].

Bradykinin can activate the B1 and B2 receptors [57]. Deleting the B2 receptor in mice increased blood pressure and cardiac mass, thus, demonstrating that bradykinin has an essential role in modulating blood pressure and cardiac remodeling [58]. Bradykinin-enhancing agents are frequently found in snake venoms. In addition, many hypotensive molecules have been isolated from *Bothrops jararaca* species, due to their ability to release bradykinin [6,7,8,9,59,60,61,62].

The molecule BmmoPLA2 was isolated from the *Bothrops moojeni* snake, the same species used in this study, and this molecule also demonstrated ability to reduce blood pressure. However, the mechanism of action has not yet been elucidated [12].

In addition to having kininogenase activity, BmooMPα-I can hydrolyze angiotensin I at the post-proline and aspartic acid positions, generating an inactive six-amino acid peptide [13]. Thus, the cleavage of angiotensin I by BmooMPα-I may also be related to the effects observed in this study.

An important point to mention is that several extracellular matrix metalloproteinases (MMPs) and ADAMs are involved in the pathophysiology of hypertension [63,64,65,66,67]. However, despite BmooMPα-I being phylogenetically related to ADAMs, this protease showed a cardioprotective effect in renovascular hypertension, possibly due to its activity of releasing bradykinin and cleaving angiotensin I [13].

This study has some limitations that must be taken into account. First, we did not assess cardiac function by echocardiography, which accurately measures systolic and diastolic function. However, we used electrocardiography to evaluate electrical conduction in the heart, which can also predict changes in ventricular dysfunction [54]. Second, we did not elucidate the mechanisms involved in blood pressure lowering and remodeling by BmooMPα-I, and this issue should be examined in further biochemical studies.

## 4. Conclusions

In conclusion, we showed that treatment with BmooMPα-I reduced blood pressure, reversed cardiac structural changes, and improved electrocardiographic changes. This response was likely mediated by kininogenase and angiotensin I cleavage activity. However, further studies are needed to elucidate the mechanism involved.

## 5. Materials and Methods

### 5.1. Venom

*Bothrops moojeni* venum was obtained from Bioactive Protein Serpentarium (Batatais, São Paulo, Brazil) and stored in the Amazon Venum Bank at CEBio (UNIR-FIOCRUZ-Rondônia). Authorization CGEN/CNPQ 010627/2011-1; IBAMA: 27131-2 and SISGEN A499B02.

### 5.2. BmooMPα-I Purification

A 50 mg sample of the *B.moojeni* venom was used to purify the metalloproteinase BmooMPα-I. The sample was resuspended in 1 mL of 50 mM ammonium bicarbonate buffer pH 8.0. The mixture was centrifuged at 13,000× *g* for 5 min, and then the supernatant was applied to a CM-SEPHAROSE FF column. The run was performed in the Akta Prime Plus Chromatography system (GE, Uppsala, Sweden) using a gradient (0–100%) of ammonium bicarbonate 500 mM pH 8.0 at a flow of 1 mL/min. The elution was monitored at 280 nm, and the fractions containing the BmooMPα-I were manually collected, lyophilized, and stored at −70 °C.

The isolated fraction, obtained by ion-exchange chromatography, was reconstituted in 0.1% Trichloroacetic acid. The mixture was applied to a C-18 column (Supelco). The reverse-phase chromatography was performed under a concentration gradient (0–70%) of acetonitrile 99.9% at a 1 mL/min flow in the Akta Prime Plus Chromatography system. Elution was monitored at 280 nm, and fractions were manually collected

### 5.3. SDS-PAGE

The presence of purified BmooMPα-I was confirmed by electrophoresis by applying 10 µg of the sample on 12% SDS-PAGE. After running, the gel was stained with Coomassie Brilliant Blue G-250, and a scanner system captured the image. The prestained protein ladder (PageRuler Plus Prestained Protein Ladder, Fermentas, Ontario, Canada) was used to confirm the molecular weight of the protein.

### 5.4. Proteolytic Activity over Casein

The proteolytic activity of the BmooMPα-I was evaluated by using 10 μg/mL of the sample that was incubated with casein 2% (*w/v*) for 30 min at 37 °C. The reaction was stopped by trichloroacetic acid 20% (*v/v*). Thus, the solution was centrifugated at 10.000 rpm for 15 min.

### 5.5. Mass Spectrometry

A sample with 50 µg of protein was treated with ammonium bicarbonate 100 mM for a total volume of 50 µL. Then, DTT 10mM was added, and the mixture was incubated at 56 °C for 60 min. Subsequently, iodoacetamide 50 mM was added and incubated for 30 min at 25 °C in the dark. Later, 200 ng/uL of trypsin was added, following overnight incubation at 37 °C. The next day, formic acid 1% was added, and the samples were dried in a speed vacuum. Finally, samples were transferred to C18 (zip Tip) for a final peptide concentration of approximately 2.5 μg/μL.

The mass analyses were performed at the Mass Spectrometry and Proteome Unit (UEMP) of the Federal University of Rio de Janeiro (UFRJ). First, samples were analyzed and ionized by electrospray. Then, after selecting the peptide in the equipment’s quadrupole, they were examined by Maldi-TOF. Finally, protein identification and de novo amino acid sequencing were performed by tandem spectrometry (MS/MS).

### 5.6. Animals

This study was carried out according to the guidelines of Ethical Principles of the National Council for the Control of Animal Experimentation (CONCEA) (CEPAE-UFPA n° 1693010319). Male Wistar rats (*n* = 36) weighing 180 g were obtained from the Central bioterium of Evandro Chagas Institute and stored in the bioterium of the Laboratory of Pharmacology and Toxicology of Natural Products. The animals were kept on a 12-h light/dark cycle at 25 °C, with free access to chow and water.

### 5.7. Experimental Design

Hypertension was induced using the model 2K1C. First, rats weighing an average of 180 g were anesthetized with ketamine 90 mg/kg and xylazine 10 mg/kg intraperitoneally. Then, after a midline laparotomy, the right renal artery was clipped with a silver clip of 0.2 mm internal diameter. Next, sham-operated rats underwent the same surgical procedure without placement of the renal artery clip. The animals were randomly assigned to six experimental groups: 1. Sham that received water (Vehicle), 2. Sham that received 1 μg/kg of BmooMPα-I; 3. Sham received 10mg/kg of Losartan; 4. 2K1C that received water (Vehicle); 5. 2K1C that received 1 μg/kg of BmooMPα-I; 6. 2K1C that received 10 mg/kg of Losartan. The 1 µg/kg dose of BmooMPα-I was based on a previous study that showed biological activity with few hemorrhagic effects [15], and the 10 mg/kg dose of Losartan was based on a previous study that demonstrated beneficial effects in lowering blood pressure and vascular remodeling in the 2K1C model of hypertension [24].

**Figure 6:**
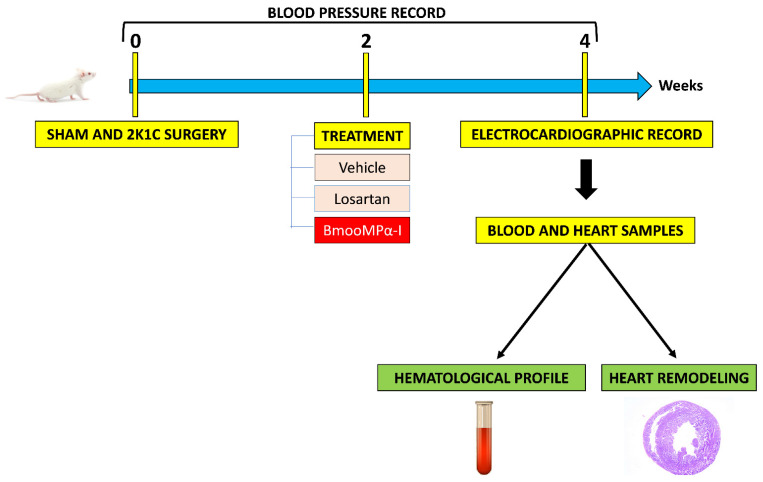
The workflow of the experimental design of the study. Hypertensive (2K1C) and normotensive (Sham) rats were treated for two weeks with Vehicle, Losartan and BmooMPα-I. Blood pressure, electrocardiographic record, hematological profile and some parameters of cardiovascular remodeling were evaluated.

The BmooMPα-I was diluted in saline and administered intraperitoneally daily, and Losartan was diluted in the Vehicle (water) and administrated by gavage daily. The treatment started in the second week and was continued until the fourth week of hypertension for another two weeks. Body weight and tail systolic and diastolic blood pressure were assessed weekly during the experimental period with a tail plethysmograph v 2.11—single channel (Insight, Ribeirão Preto, Brazil). Figure 6 represents a workflow of the experimental design of the study.

### 5.8. Electrocardiographic Record

The animals from the interest groups were submitted to the ECG in the fourth week of hypertension, recording six animals from each group. First, the animals were anesthetized with tribromoethanol 15 mL/kg [68] and carefully shaved in the thoracic region before recording the ECG. Then, the animal was carefully positioned inside a Faraday cage at the recording time. Then, three electrodes were fixed, the first ground electrode on the animal’s hind paw, the second reference electrode on the right shoulder and the third electrode on the opposite side to the left of the xiphoid process in the DII lead. All were made of silver measuring 7 × 3 mm and connected directly to an acquisition system. A differential amplifier performed the record acquisition with high-impedance AC input (Grass Technologies, P511, West Warwick, Rhode Island, USA), adjusted with filtering of 0.3 Hz (high-pass) and 300 Hz (low-pass). The records were monitored with an oscilloscope (Protek, 6510) and continuously digitized at 10 kHz by a computer equipped with Axoscope 9.0 software (Axon Instruments, Champaign, IL, USA), recording time was 5 min for each animal, and the files being saved and stored for later analysis. The records were analyzed using the “LabChart v.7.3.8” interface software (ADInstruments, Colorado Springs, CO, USA). A part of the 180-s electrocardiographic tracing was randomly selected, which contained an R-R interval sequence, which were used to calculate the heart rate (bpm), QRS complex and PR, and QT segments [69,70,71]. The QTc was calculated using Bazett’s formula with modifications for rodents, QTc = QT/√(RR/150) [68].

### 5.9. Sample Collection and Histological Preparation of Hearts

In the fourth week of hypertension, the animals were weighed, anesthetized, and their thoracic cavity was opened to expose the beating heart. First, a whole blood sample was taken and stored in a disodium EDTA tube. Then, the heart was rapidly removed, rinsed in 0.9% saline solution, and placed in chloride potassium 50 mM to maintain the diastole phase. Posteriorly, the heart was weighed and fixed as a whole in phosphate buffer 10% formalin (pH 7.4). Next, both ventricles from the heart were isolated and cut into two fragments by a mid-ventricular coronal section. Then, each block was serially cut in the same direction, and 4-μm thick sections were stained with hematoxylin and eosin and picrosirius.

### 5.10. Serum Measurement of Blood Cells

The samples were analyzed using a semi-automatic methodology, using the pocH-100iV counter (Sysmex), in which the amount of hemoglobin, red and white blood cells and platelets was quantified.

### 5.11. Heart Morphometry, Myocyte Diameter and Collagen Content

The first three HE-stained histological sections determined the left ventricular wall thickness, interventricular septum, and left ventricular chamber area. The sections were photographed using a Leica M205 loupe using the Leica DFC450 camera, and the analyzes were performed using the Leica application Suite software.

Myocytes were measured using HE-stained sections photographed at 400x magnification under a white light microscope (Carl Zeiss Microscopy Ltd., Cambridge, UK). In total, 10 fields were photographed per slide, where 10 myocytes were quantified per field, totaling 100 myocytes per animal. The myocyte diameter was obtained in the region of the cardiac fiber, where the nucleus was well-defined, and it was possible to identify the ends of the cardiac fiber. Analyzes were performed using Image J 64-bit software from the National Institutes of Health (NIH). The image with a scale was opened in the Image J software. Then, the straight line option was selected to measure the size of the scale present in the picture. The “set scale” option was selected, where the known distance is informed.

Interstitial collagen content was measured using Picrosirius stained sections photographed at 400× magnification under a white and polarized light microscope (Carl Zeiss Microscopy Ltd., Cambridge, UK). In total, 10 randomly chosen fields per animal were photographed and quantified. Analyzes were performed using Image J 64-bit software from the National Institutes of Health (NIH). The same image was opened twice in Image J, and one was converted to 16 bits. Then, in the 16-bit picture, the threshold adjustment option was selected. The marked area was adjusted based on the first image. Subsequently, the particle analysis action was chosen to obtain the area marked for collagen.

### 5.12. Statistical Analysis

The results are expressed as mean ± SD for the *measurement of blood cells*; for the other tests, we used mean ± S.E.M. The data were analyzed on Graph Pad Prism^®^ 8.0 software (Graph Pad, San Diego, CA, USA). Data normality was assessed with the Shapiro–Wilk test. The values were considered normal. Therefore, we performed Two-way ANOVA followed by the Tukey test. The results were considered statistically significant when *p* ≤ 0.05.

## Figures and Tables

**Table 1:** Hematological profile of Sham and 2K1C rats treated with BmooMPα-I and Losartan.

	Erythrocyte (×10^6^/μL)	Hemoglobin (g/dL)	Platelets (×10^3^/μL)	Leucocytes (×10^6^/μL)
Sham Vehicle	7.2 ± 0.4	13 ± 0.6	725 ± 73	4883 ± 1156
Sham BmooMPα-I	8.1 ± 0.4	13 ± 0.5	693 ± 34	4583 ± 647
Sham Losartan	7.8 ± 0.8	14 ± 0.2	695 ± 71	4983 ± 938
2K1C Vehicle	7.9 ± 0.3	13 ± 0.3	726 ± 75	4400 ± 800
2K1C BmooMPα-I	8.2 ± 0.5	13 ± 1.1	758 ± 67	3980 ± 326
2K1C Losartan	8.3 ± 1.2	13 ± 2.4	681 ± 111	4316 ± 609

Data are shown as mean ± SD (n = 6/group). *p* > 0.05.

**Table 2:** Electrocardiographic parameters of Sham and 2K1C rats with their respective treatments.

	Hearth Rate (bpm)	PR (ms)	QRS (ms)	QT (ms)	QTc (ms)
Sham Vehicle	302 ± 2	50 ± 0.1	36 ± 0.4	89 ± 2	79 ± 3
Sham BmooMPα-I	297 ± 3	50 ± 0.2	36 ± 0.1	90 ± 1	78 ± 7
Sham Losartan	291 ± 8	49 ± 0.2	40 ± 0.1	91 ± 1	80 ± 9
2K1C Vehicle	332 ± 6 *	52 ± 0.1	48 ± 0.2 *	122 ± 1 *	126 ± 2
2K1C BmooMPα-I	297 ± 4 ^#^	50 ± 0.1	42 ± 0.1 ^#^	92 ± 1 ^#^	80 ± 4
2K1C Losartan	300 ± 5 ^#^	51 ± 0.2	36 ± 0.1 ^#^	89 ± 2 ^#^	80 ± 5

bpm (beats per minute), ms (milliseconds). Data are shown as mean ± S.E.M. (n = 6/group). * *p* < 0.05 versus Sham Vehicle group and ^#^ *p* < 0.05 versus 2K1C Vehicle group.

**Table 3:** Parameters of cardiac remodeling of Sham and 2K1C rats treated with BmooMPα-I and Losartan.

	HW/BW Ratio (mm/g)	LVWT (mm)	ISWT (mm)	LVCA (mm^2^)	Myocyte Diameter (µm)	Collagen Content (% Area)
Sham Vehicle	3.0 ± 0.1	2.4 ± 0.04	2.1 ± 0.02	12.1 ± 0.5	4.4 ± 0.1	4.1 ± 0.3
Sham BmooMPα-I	3.2 ± 0.1	2.4 ± 0.04	2.2 ± 0.03	11.8 ± 0.5	4.6 ± 0.1	4.2 ± 0.3
Sham Losartan	3.0 ± 0.2	2.4 ± 0.03	2.3 ± 0.02	12.4 ± 0.6	4.3 ± 0.1	4.1 ± 0.2
2K1C Vehicle	3.6 ± 0.1 *	2.8 ± 0.05 *	2.5 ± 0.04 *	10.5 ± 0.3 *	6.0 ± 0.1 *	7.4 ± 0.8 *
2K1C BmooMPα-I	3.2 ± 0.1 ^#^	2.5 ± 0.02 ^#^	2.3 ± 0.06 ^#^	12.1 ± 0.3 ^#^	4.9 ± 0.1 ^#^	3.9 ± 0.3 ^#^
2K1C Losartan	3.0 ± 0.1 ^#^	2.5 ± 0.06 ^#^	2.2 ± 0.02 ^#^	12.2 ± 0.4 ^#^	4.8 ± 0.1 ^#^	4.4 ± 0.3 ^#^

HW/BW (heart weight/body weight), LVWT (left ventricle wall thickness), ISWT (interventricular septum wall thickness). LVCA (left ventricle chamber area). Data are shown as mean ± S.E.M. (n = 6/group). * *p* < 0.05 versus Sham Vehicle group and ^#^ *p* < 0.05 versus 2K1C Vehicle group.

## Data Availability

Not applicable.

## Supplementary Materials

The following supporting information can be downloaded at: https://www.mdpi.com/article/10.3390/toxins14110766/s1, Figure S1. Image original of the Gel SDS-PAGE of the fraction isolated on reversed-phase column showing a single band of the purified protein corresponding to BmooMPα-I.
