# Supplementary material for: BmooMPα-I, a Metalloproteinase Isolated from Bothrops moojeni Venom, Reduces Blood Pressure, Reverses Left Ventricular Remodeling and Improves Cardiac Electrical Conduction in Rats with Renovascular Hypertension
Source: Toxins (Basel). 2022 Nov 5;14(11):766. doi: 10.3390/toxins14110766 (PMC9697896; doi:10.3390/toxins14110766)
Supplement: Supplementary file 1 [file toxins-14-00766-s001.zip › toxins-1899460-supplementary.pdf]

toxins

Supplementary Material: BmooMPα-I, a Metalloproteinase Isolated from *Bothrops moojeni* Venom, Reduces Blood Pressure, Reverses Left Ventricular Remodeling and Improves Cardiac Electrical Conduction in Rats with Renovascular Hypertension

Jorge Eduardo Chang, Keuri Eleutério Rodrigues, Anderson Maciel, Cahy Manoel Bannwart, Wictória Farias Dias, Moisés Hamoy, Russolina Benedeta Zingali, Andreimar Martins Soares, Carolina Heitmann Mares Azevedo Ribeiro, Raquel Fernanda Gerlach, Marta Chagas Monteiro and Alejandro Ferraz Prado

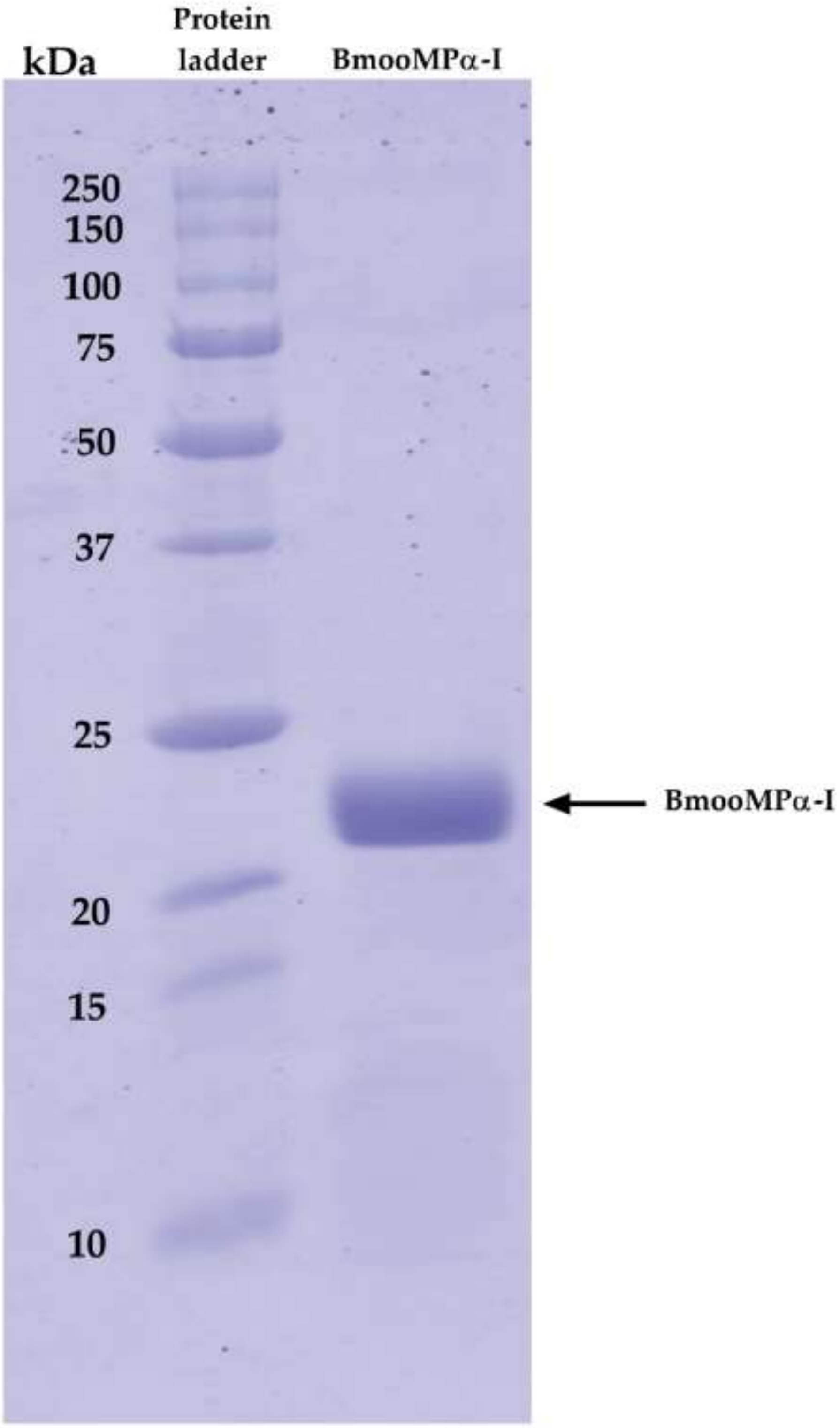

Figure S1. Image original of the Gel SDS-PAGE of the fraction isolated on reversed-phase column showing a single band of the purified protein corresponding to BmooMPα-I.
